# Level I of evidence does not support manual lymphatic drainage for total knee arthroplasty: a meta-analysis

**DOI:** 10.1038/s41598-023-49291-y

**Published:** 2023-12-12

**Authors:** Filippo Migliorini, Luise Schäfer, Francesca Alzira Bertini, Michael Kurt Memminger, Francesco Simeone, Riccardo Giorgino, Nicola Maffulli

**Affiliations:** 1 Department of Orthopaedic, Trauma, and Reconstructive Surgery, RWTH University Medical Centre, Pauwelsstraße 30, 52074 Aachen, Germany; 2 Department of Orthopedics and Trauma Surgery, Academic Hospital of Bolzano (SABES-ASDAA), Teaching Hospital of the Paracelsus Medical University, 39100 Bolzano, Italy; 3 Residency Program in Orthopedics and Traumatology, University of Milan, Milan, Italy; 4 Department of Medicine and Psychology, University of Rome “La Sapienza”, Rome, Italy; 5 School of Pharmacy and Bioengineering, Keele University Faculty of Medicine, Stoke on Trent, ST4 7QB UK; 6 Centre for Sports and Exercise Medicine, Barts and the London School of Medicine and Dentistry, Mile End Hospital, Queen Mary University of London, London, E1 4DG UK

**Keywords:** Health care, Medical research

## Abstract

It is unclear whether manual lymphatic drainage (MLD) following primary total knee arthroplasty (TKA) is effective in reducing pain and swelling and improving knee function. The present study investigated the efficacy of MLD after TKA. The outcomes of interest are the range of motion (ROM), pain (visual analogue scale, VAS), and circumference of the lower leg. This meta-analysis was conducted according to the 2020 PRISMA statement. In November 2023, the following databases were accessed: PubMed, Web of Science, Google Scholar, and Embase, with no time constraint. Only level I evidence studies, according to the Oxford Centre of Evidence-Based Medicine, were considered. All the randomised controlled trials (RCTs) comparing patients who have received MLD versus a group of patients who did not undergo MLD following primary TKA were accessed. Data from four RCTs (197 TKAs) were retrieved. 67% (132 of 197 patients) were women. The mean length of follow-up was 7.0 ± 5.8 weeks. The mean age of the patients was 69.6 ± 2.7 years, and the mean BMI was 28.7 ± 0.9 kg/m^2^. At baseline, between-group comparability was evidenced in the male:female ratio, mean age, mean BMI, knee flexion, and VAS. No difference was found in flexion (P = 0.7) and VAS (P = 0.3). No difference was found in the circumference of the thigh (P = 0.8), knee (P = 0.4), calf (P = 0.4), and ankle (P = 0.3). The current level I of evidence does not support the use of MLD in primary TKA.

## Introduction

In patients with end-stage osteoarthritis^1,2^, total knee arthroplasty (TKA) is commonly performed^3–5^. TKA restores joint function, improving the quality of life and participation in recreational activities^6–11^. In the past few decades, the number of patients undergoing TKAs has increased, and the design and techniques also developed quickly to ensure the highest standards^12–17^. Postoperative rehabilitation to favor and embrace recovery following TKA is debated^18,19^. Several studies investigated methods to improve function and shorten the time to full recovery, starting rehabilitation in the first postoperative days^20–26^. In this context, manual lymphatic drainage (MLD) has been advocated in this phase of postoperative recovery to reduce oedema and pain^27–29^. The principle behind MLD is the stimulation and improvement of the lymphatic system, promoting variations in interstitial pressures through the application of gentle pressure^27,28^. Regarding the potential effect of MLD on pain, the exact mechanism is not yet clear, and the placebo effect may play a major role^30,31^. However, whether MLD is effective in reducing pain and swelling and improving knee function is still unclear and evidence is controversial. Recently, four randomised controlled trials (RCTs) which evaluated the efficacy of MLD in TKA have been published^32–35^; however, to the best of our knowledge, an updated meta-analysis is missing.

The present study investigated the efficacy of MLD after TKA. The outcomes of interest are the range of motion (ROM), pain, and circumference of the lower leg.

## Methods

### Eligibility criteria

All the clinical studies comparing MLD versus a group of patients who did not undergo MLD following TKA were accessed. Only studies published in peer-reviewed journals were considered. According to the author’s language capabilities, articles in English, German, Italian, French, and Spanish were eligible. Only level I of evidence studies, according to the Oxford Centre of Evidence-Based Medicine^36^, were considered. Studies which evaluated arthroplasty in other body areas were not considered, nor were those conducted in TKA revision settings. Reviews, opinions, letters, and editorials were not considered. Missing quantitative data under the outcomes of interests warranted the exclusion of the study. Only studies that clearly stated the sample size were considered.

### Search strategy

This study was conducted according to the Preferred Reporting Items for Systematic Reviews and Meta-Analyses: the 2020 PRISMA statement^37^. The PICOD algorithm was preliminarily established:

1. P (Problem): postoperative recovery in TKA.
2. I (Intervention): MLD.
3. C (Control): non-MLD.
4. O (Outcomes): ROM, pain, and swelling.
5. D (Design): RCT.

In November 2023, the following databases were accessed: PubMed, Web of Science, Google Scholar, and Embase. No time constraint was set for the search. The Medical Subject Headings (MeSH) used for the database search are reported as supplementary material. The search was restricted to RCTs.

### Selection and data collection

Selection and data collection were performed by two authors (L.S. & R.G.). All the titles resulting from the literature search were screened by hand and the abstract of those which matched the topic were accessed. If the abstract matched the topic, the full text was accessed. Moreover, the same authors conducted a cross-reference of the bibliography of the full texts for inclusion. A third senior author (N.M.) took the final decision in case of divergencies.

### Data items

Two authors (L.S. & R.G.) performed data extraction. The following data at baseline were extracted: author, year of publication and journal, length of the follow-up, number of patients with related mean age, and BMI. Data concerning knee flexion and Visual Analogue Scale (VAS) were collected at baseline and last follow-up. Data on the circumference of the lower leg (tight, knee, calf, ankle) at last follow-up were collected. Data were extracted in Microsoft Office Excel version 16.72 (Microsoft Corporation, Redmond, USA).

### Assessment of the risk of bias and quality of the recommendations

The risk of bias was evaluated following the guidelines Cochrane Handbook for Systematic Reviews of Interventions^38^. One reviewer (L.S.) evaluated the risk of bias in the extracted studies using the risk of bias assessment tool (RoB2)^39,40^ of the software Review Manager 5.3 (The Nordic Cochrane Collaboration, Copenhagen). The following endpoints were evaluated: bias arising from the randomisation process, bias from deviations from intended interventions, bias from missing outcome data, bias in measurement of the outcome, and bias in selection of the reported result.

### Synthesis methods

The statistical analyses were performed by the main author (F.M.) following the recommendations of the Cochrane Handbook for Systematic Reviews of Interventions^41^. To assess data distribution the Saphiro–Wilk test was used. For parametric data, the mean value and standard deviation (SD) were calculated. For non-parametric data, the median value and interquartile range (IQR) were evaluated. To assess baseline comparability, the unpaired t-test for parametric data or the Mann–Whitney test for non-parametric variables were used. The meta-analyses were conducted using the software Review Manager 5.3 (The Nordic Cochrane Collaboration, Copenhagen). For continuous data, the inverse variance method with mean difference (MD) effect measure was used. For dichotomic data, the Mantel–Haenszel method with odd ratio (OR) effect measure was used. The CI was set at 95% in all the comparisons. Heterogeneity was evaluated through Higgins-I^2^ and χ^2^ tests. If P_χ2_ > 0.05, no statistically significant heterogeneity was found. If P_χ2_ < 0.05, the heterogeneity was estimated using the Higgins-I^2^ as follows: low (< 30%), moderate (30% to 60%), and high (> 60%). A fixed effect model was set as default. If moderate or high heterogeneity was detected, a random model effect was used. Overall values of P < 0.05 were considered statistically significant.

### Ethical approval

This study complies with ethical standards.

## Results

### Study selection

**Figure 1:**
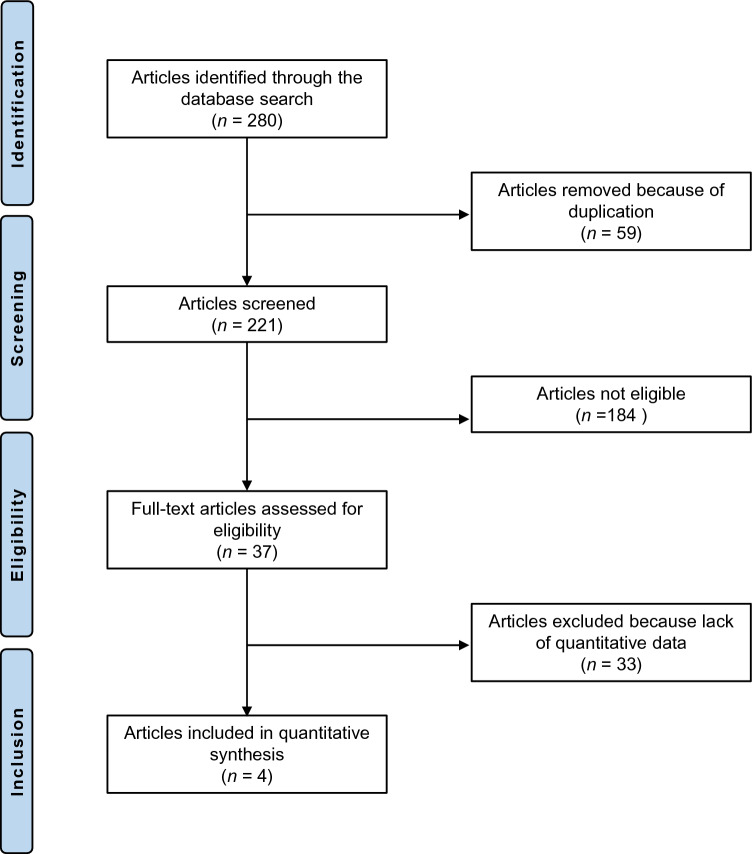
PRISMA flow chart of the literature search.

The systematic literature search resulted in 280 articles. A total of 59 duplicates were identified and therefore removed. After reviewing the abstracts of the remaining 221 studies, a further 184 articles were discarded because they did not match the eligibility criteria. The detailed reasons leading to exclusion were: study type and design (*N* = 108), low level of evidence (*N* = 53), full-text not available (*N* = 5), and language limitations (*N* = 18). An additional 33 studies missed quantitative data under the outcomes of interest and were therefore not considered. In conclusion, four RCTs were included in the present meta-analysis. The results of the literature search are shown in Fig. 1.

### Risk of bias assessment

**Figure 2:**
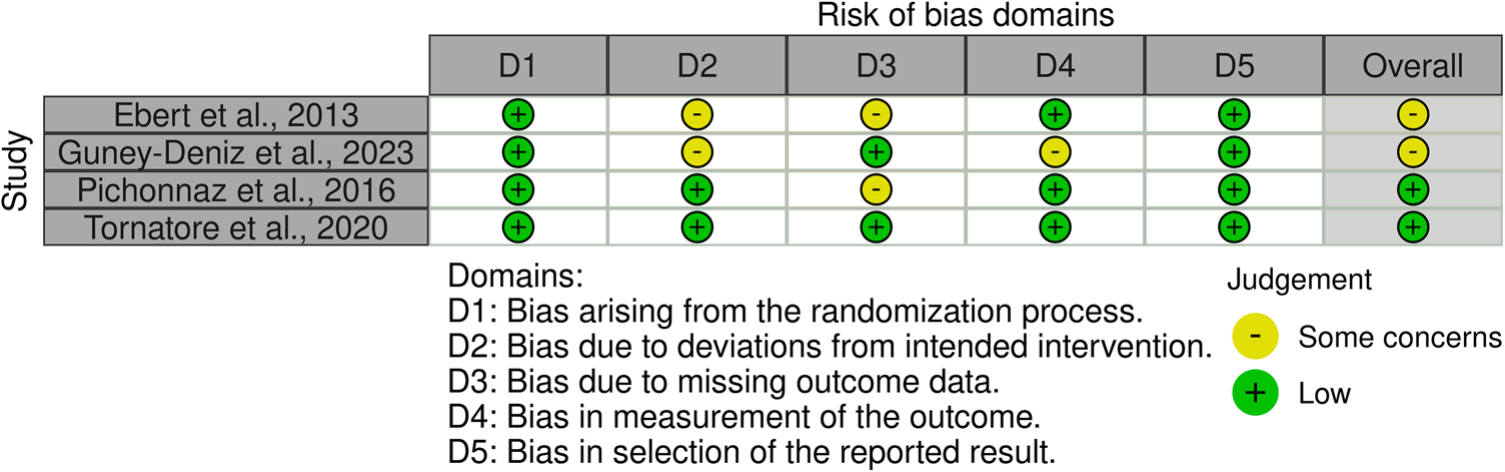
Cochrane risk of bias tool (ROB2).

The Cochrane risk of bias tool (ROB2) was used to investigate the risk of bias in all studies included in the present meta-analysis. The randomisation process was predominantly of high quality using a random number generator. No baseline differences were found between study groups in any study. In two studies, there were concerns about deviations from the planned intervention because patients and investigators were not blinded after randomisation. The risk of bias from missing outcome data was noted in two studies, as the number of patients who dropped out during the study period differed between the comparison groups and was not compensated for. In terms of outcome measurement, only one study raised concerns, and the risk of bias in the selection of the reported outcome was uniformly low. Concluding, the risk of bias graph evidenced a predominantly low to moderate quality of the methodological assessment of the RCTs (Fig. 2).

### Study characteristics and results of individual studies

Data from 197 TKAs were retrieved. 67% (132 of 197 patients) were women. The mean length of the follow-up was 7.0 ± 5.8 weeks. The mean age of the patients was 69.6 ± 2.7 years, and the mean BMI was 28.7 ± 0.9 kg/m^2^. The generalities and demographics of the included studies are shown in Table 1.

**Table 1:** Generalities and patient baseline of the included studies (*MLD* manual lymphatic drainage).

Author (year)	Journal	Follow-up (weeks)	Group	Patients (*n*)	Women (*n*)	Mean age	Mean BMI (kg/m^2^)
Ebert et al. (2013)^32^	Arch Phys Med Rehabil	6	MLD	24	7	70.8	28.2
			Control	26	7	69.2	27.7
Guney-Deniz et al. (2023)^33^	Physiother Theory Pract	6	MLD	13	13	65.5	27.8
			Control	15	15	65.4	29.1
Pichonnaz et al. (2016)^34^	Arch Phys Med Rehab	15	MLD	29	18	71.3	28.2
			Control	24	21	70.1	29.9
Tornatore et al. (2020)^35^	Int J Rehabil Res	1	MLD	33	26	71.3	28.96
			Control	33	25	72.82	29.88

### Baseline comparability

At baseline, between-group comparability was evidenced in the ratio of male:female, mean age, mean BMI, knee flexion, and VAS (Table 2).

**Table 2:** Baseline comparability (*MLD* manual lymphatic drainage, *VAS* visual analogue scale).

Endpoint	MLD (n = 99)	Non-MLD (n = 98)	P
Women	68% (64 of 99)	73% (68 of 98)	0.8
Mean age	69.7 ± 2.8	69.4 ± 3.1	0.9
Mean BMI	28.3 ± 0.5	29.1 ± 1.0	0.2
Flexion (°)	125.3 ± 1.7	126.2 ± 1.6	0.6
VAS (0–10)	5.5 ± 0.9	5.2 ± 0.8	0.7

### Synthesis of results

**Figure 3:**
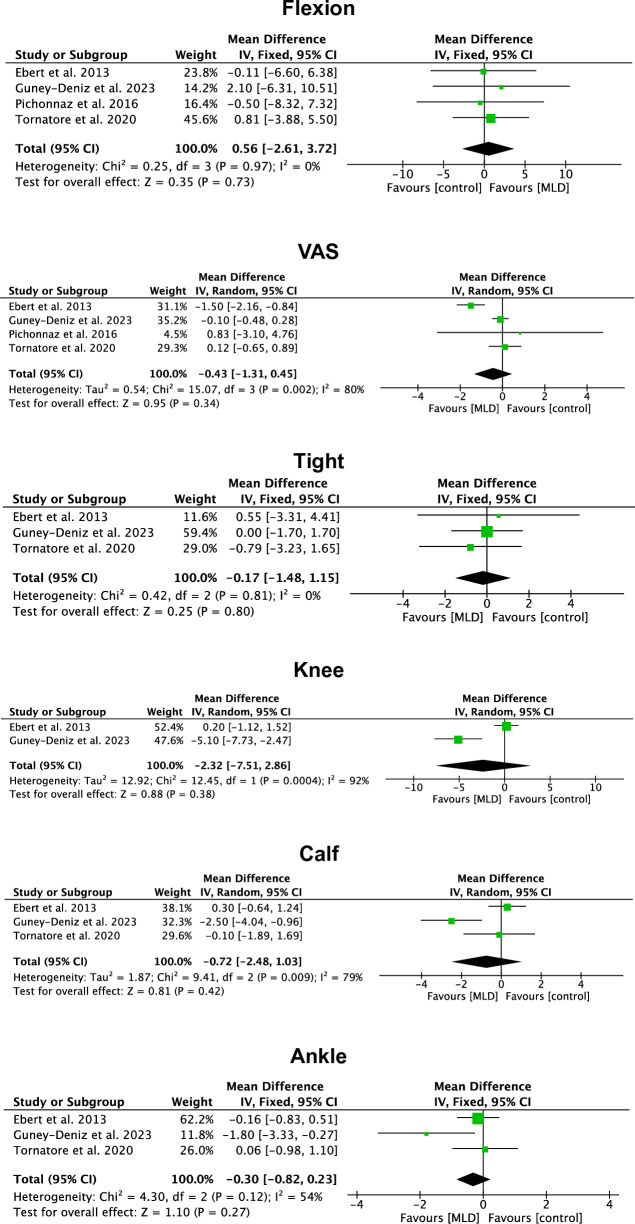
Forest plots of the meta-analyses (*VAS* visual analogue scale).

No difference was found in flexion (P = 0.7) and VAS (P = 0.3). No difference was found in the circumference of the lower leg: tight (P = 0.8), knee (P = 0.4), calf (P = 0.4), and ankle (P = 0.3). The forest plots are reported in Fig. 3.

## Discussion

According to the findings of the present meta-analysis, the current level I of evidence does not support the use of MLD in primary TKA.

Ebert et al.^32^ randomised 41 patients (50 knees) to receive MLD or to the control group. Patients who underwent MLD demonstrated significantly greater active knee flexion 4 days and 6 weeks after TKA^32^. However, no significant effects were found on passive knee flexion, lower limb circumference, or subjective scores^32^. In the RCT by Guney-Deniz et al.^33^, 45 female patients with unilateral TKA were assigned to MLD (n = 15) combined with exercises, kinesiotaping (n = 15) combined with exercises, or exercises in isolation (n = 15). MLD significantly reduced pain and thigh and calf oedema from the second to the fourth postoperative day, with greater efficacy compared to the control group^33^. These results were confirmed also 2 weeks postoperatively^33^. No difference was found in the range of motion^33^. At 6-week follow-up, no differences were observed between the groups in oedema, pain, motion, and patient-reported outcome measures (PROMs)^33^. Pichonnaz et al.^34^ randomly allocated 60 patients to receive five sessions of MLD in addition to the usual postoperative rehabilitation or to the control group. There was no significant difference between the two groups at 7 days and 3 months, except for passive knee flexion contracture at 3 months, which was lower and less frequent in the MLD group^34^. Pain levels significantly decreased after MLD treatments^34^. MLD was not effective in reducing oedema^34^. In another RCT on 99 patients, Tornatore et al.^35^ compared kinesiotaping combined with MDL vs. MDL or kinesiotaping in isolation. Combined kinesiotaping and MLD reduced pain and oedema more than any other treatment in isolation^35^. MLD in isolation was more effective in reducing oedema than kinesiotaping in isolation^35^. No differences were found in flexion among the groups^35^.

The present investigation did not identify clinical advantages of MLD in TKA. The duration and intensity of MLD sessions were variable among the included studies, which may have affected the validity of the results. The techniques and MLD protocols were not described in depth, which represents another important limitation, which may have impacted the reliability of the present results. Standardizing MLD protocols, that provide adequate descriptions of the technique would provide more consistent and comparable data for analysis in future research. Irrespective of these limitations, results from the present study are consistent with the current literature. The efficacy of MLD in musculoskeletal medicine is controversial^42–46^. After orthopaedic surgery, tissue swelling may be associated with a longer recovery and increased pain, limited mobility, reduced function, and interference with the wound healing process^47,48^. The most important limitation on the clinical efficacy of MLD is the limited evidence available in the current literature. Moreover, considering the lack of guidelines or recommendations, the use of MLD following TKA should be considered cautiously. MLD has been evaluated also in other areas, with similar conclusions^49–52^.

Additional limitations are evident. There was much variability between the included studies in sample size, duration of follow-up, intervention protocols, and outcome measures. Another major limitation of the present study is the lack of standardisation of MLD protocols and techniques in terms of session duration, intensity, and frequency, which could have led to potential variations in the efficacy of the intervention. Moreover, the surgical technique, exposure, and implants were not often described. Similarly, whether patients underwent patellar resurfacing or received cemented or press-fit implants was often biased, as was the use of the tourniquet or tranexamic acid. Given the lack of quantitative data, additional analyses were not possible. In the outcome assessment, the measures were limited to flexion, pain, and lower leg circumference. Other relevant outcomes, such as function, quality of life, and patient satisfaction PROMs, should be considered in future investigations. An additional implication of MLD is the evaluation of cost efficacy, as this procedure requires trained personnel. The method of MLD was not specified in detail in most studies. Being a non-invasive procedure, MLD deserves further research to provide more robust evidence in support of its efficacy in TKA. The risk of bias graph evidenced a predominantly low to moderate quality of the methodological assessment of the RCTs. In two studies, there were concerns about deviations from the planned intervention because patients and investigators were not blinded after randomisation. The risk of bias from missing outcome data was noted in two studies, as the number of patients who dropped out during the study period differed between the comparison groups and was not compensated for. In terms of outcome measurement, only one study raised concerns, and the risk of bias in the selection of the reported outcome was uniformly low. More precisely, in the study conducted by Ebert et al.^32^, all in-hospital clinical assessments were carried out by physiotherapists who were unaware of patient randomization. However, despite preventing the supervising physiotherapist from having contact with massage therapists and instructing patients to avoid discussing study information with their supervising physiotherapist, achieving complete blinding in a study of this nature proved to be difficult. In the study by Pichonnaz et al.^34^, the measurement of pain was performed by the treating physiotherapist immediately after the treatment. This aspect represents a lack of blinding that could have influenced the results (Supplementary Information).

## Conclusion

According to the current level I evidence, MLD in primary TKA is not associated with an improvement in ROM, pain, and circumference of the lower leg. Additional high-quality investigations are strongly required to assess the efficacy of MLD in primary TKA.

### Supplementary Information

Supplementary Information.

## Data Availability

The datasets generated during and/or analysed during the current study are available throughout the manuscript.
