# Supplementary material for: Level I of evidence does not support manual lymphatic drainage for total knee arthroplasty: a meta-analysis
Source: Sci Rep. 2023 Dec 12;13:22024. doi: 10.1038/s41598-023-49291-y (PMC10716409; doi:10.1038/s41598-023-49291-y)
Supplement: Supplementary file 1 — Supplementary Information. [file 41598_2023_49291_MOESM1_ESM.docx]

**Systematic literature search for:**

# Kinesio Taping^®^ for total knee arthroplasty: a level I evidence-based meta-analysis

**Concept 1: Total Knee Arthroplasty (TKA)**

**Keywords:**

Total Knee Arthroplasty

Primary Total Knee Arthroplasty

TKA

**Mesh:**

"Surgical Procedures, Operative"[Mesh]

"Orthopedic Procedures"[Mesh]

"Knee Joint/surgery"[Mesh]

"Arthroplasty"[Mesh]

"Arthroplasty, Replacement, Knee"[Mesh]

"Arthroplasty, Replacement, Knee/methods*"[Mesh]

"Arthroplasty, Replacement, Knee / adverse effects"[Mesh]

"Knee Prosthesis"[Mesh]

"Prosthesis Implantation"[Mesh]

**Concept 2: Therapy**

**Keywords:**

Manual lymphatic drainage

MLD

Postoperative Period

Postoperative care

Postoperative care/*methods

Recovery of Function

Treatment Outcome

Quality of Life

Range of Motion

Range of Motion, Articular

Pain

*Pain Management

Pain Measurement

**Mesh:**

"Musculoskeletal Manipulations"[Mesh]

"Complementary Therapies"[Mesh]

"Manual Lymphatic Drainage"[Mesh]

"Postoperative Period"[Mesh]

"Postoperative Care"[Mesh]

"Postoperative Care/methods"[Mesh]

"Recovery of Function"[Mesh]

"Recovery of Function/physiology"[Mesh]

"Treatment Outcome"[Mesh]

"Quality of Life"[Mesh]

"Range of Motion, Articular"[Mesh]

"Range of Motion, Articular/physiology"[Mesh]

"Pain"[Mesh]

"Pain Management"[Mesh]

"Pain Measurement"[Mesh]

**Concept 1: Total Knee Arthroplasty (TKA)**

"Surgical Procedures, Operative"[Mesh] OR "Orthopedic Procedures"[Mesh] OR "Arthroplasty"[Mesh] OR "Arthroplasty, Replacement, Knee"[Mesh] OR "Arthroplasty, Replacement, Knee/adverse effects"[Mesh] OR "Arthroplasty, Replacement, Knee/methods"[Mesh] OR "Knee Prosthesis"[Mesh] OR "Prosthesis Implantation"[Mesh] OR Total Knee Arthroplasty OR Primary Total Knee Arthroplasty OR TKA

**AND**

**Concept 2: Therapy**

"Musculoskeletal Manipulations"[Mesh] OR "Complementary Therapies"[Mesh] OR "Manual Lymphatic Drainage"[Mesh] OR Manual lymphatic drainage OR MLD

**AND**

"Postoperative Period"[Mesh] OR "Postoperative Care"[Mesh] OR "Postoperative Care/methods"[Mesh] OR "Recovery of Function"[Mesh] OR "Recovery of Function/physiology"[Mesh] OR "Treatment Outcome"[Mesh] OR "Quality of Life"[Mesh] OR Postoperative Period OR Postoperative care OR Postoperative care/*methods OR Recovery of Function OR Treatment Outcome OR Quality of Life

**AND**

"Range of Motion, Articular"[Mesh] OR "Range of Motion, Articular/physiology"[Mesh] OR Range of Motion OR Range of Motion, Articular

**AND**

"Pain"[Mesh] OR "Pain Management"[Mesh] OR "Pain Measurement"[Mesh] OR Pain OR *Pain Management OR Pain Measurement

**Summary of the Search**

(((("Surgical Procedures, Operative"[Mesh] OR "Orthopedic Procedures"[Mesh] OR "Arthroplasty"[Mesh] OR "Arthroplasty, Replacement, Knee"[Mesh] OR "Arthroplasty, Replacement, Knee/adverse effects"[Mesh] OR "Arthroplasty, Replacement, Knee/methods"[Mesh] OR "Knee Prosthesis"[Mesh] OR "Prosthesis Implantation"[Mesh] OR Total Knee Arthroplasty OR Primary Total Knee Arthroplasty OR TKA) AND ("Musculoskeletal Manipulations"[Mesh] OR "Complementary Therapies"[Mesh] OR "Manual Lymphatic Drainage"[Mesh] OR Manual lymphatic drainage OR MLD)) AND ("Postoperative Period"[Mesh] OR "Postoperative Care"[Mesh] OR "Postoperative Care/methods"[Mesh] OR "Recovery of Function"[Mesh] OR "Recovery of Function/physiology"[Mesh] OR "Treatment Outcome"[Mesh] OR "Quality of Life"[Mesh] OR Postoperative Period OR Postoperative care OR Postoperative care/*methods OR Recovery of Function OR Treatment Outcome OR Quality of Life)) AND ("Range of Motion, Articular"[Mesh] OR "Range of Motion, Articular/physiology"[Mesh] OR Range of Motion OR Range of Motion, Articular)) AND ("Pain"[Mesh] OR "Pain Management"[Mesh] OR "Pain Measurement"[Mesh] OR Pain OR *Pain Management OR Pain Measurement)
